# Management of a patient with thalamic-capsular hematoma complicated with perilesional edema: a case report

**DOI:** 10.3389/fnhum.2025.1559631

**Published:** 2025-07-02

**Authors:** Richa Gandhi, Anshu Tikariha, Shivani Dey, Pooja Ladkhedkar

**Affiliations:** Department of Neurophysiothery, Ravi Nair Physiotherapy College, Datta Meghe Institute of Higher Education and Research, Wardha, India

**Keywords:** intermittent theta burst stimulation, thalamic hematoma, perilesional edema, multimodal stimulation, physiotherapy management

## Abstract

The patient is a 45-year-old woman who was rehabilitated from perilesional oedema with a right thalamic-capsular hematoma. Functional dependence and left-sided hemiparesis were noted. There was considerable improvement in mobility, strength and independence after providing holistic physiotherapy that consisted of multimodal sensory stimulation, tone management, and functional training. Recovery was further enhanced with cutting-edge treatments, including intermittent theta burst stimulation. Remarkable improvements were evidenced in muscle strength, cognitive orientation, and functional independence measures over the entire course of treatment. The article illustrates how quickly and effectively crucial oriented physical therapy at the right point can help reach the best benefit outcome by using the case scenario of a thalamic patient.

## Introduction

Thalamic-capsular hematoma with perilesional edema is a significant medical condition characterized by bleeding within the thalamus and the surrounding internal capsule, often accompanied by oedema (swelling) in the adjacent brain tissue (Renard et al., 2014). Thalamic haemorrhage is one of the most common types of nontraumatic intracerebral haemorrhages, accounting for 10 to 15% of all cases (Ruiz-Sandoval et al., 2019). Cerebral haemorrhage occurs in 18.5% of stroke patients, and thalamic haemorrhage accounts for 26% of all cerebral haemorrhage (Hiraoka et al., 2017). Thalamocapsular hematomas are most commonly caused by chronic hypertension, vascular malformations, and cerebral amyloid angiopathy (Qureshi et al., 2001). Other contributing factors include anticoagulant therapy, coagulopathies, trauma, substance abuse, infections, vasculitis, and haemorrhagic brain tumors. Understanding these aetiologies is crucial for guiding management and improving outcomes (Flaherty et al., 2007).

Depending on which parts of the thalamus and internal capsule are involved, common symptoms of a thalamic-capsular haematoma might vary but typically include weakness. Hemiparesis, or weakness on one side of the body, is common in patients and can affect daily tasks and movement. Sensory disturbances include alterations in touch, pain, temperature, and pressure perception, usually affecting one side of the body, and the absence or abnormality of these feelings. Cognitive deficits, including confusion, memory loss, and issues with judgment or decision-making and reasoning are some of the symptoms. Modifications in behaviour: because the thalamus regulates emotions, depression, agitation, and variations in motivation or interest in activities are common which are due to thalamic involvement (Umphred et al., 2013; Roh et al., 2021; Arboix et al., 2007).

Perilesional oedema (PHE) is recognized as a quantifiable marker of secondary brain injury following intracerebral haemorrhage (ICH). Its presence is correlated with poor outcomes, indicating that the extent of oedema can reflect the severity of brain injury and the potential for recovery (Chung et al., 1996). Research indicates that patients with thalamic strokes who receive timely physiotherapy interventions demonstrate better recovery trajectories compared to those who do not. For instance, a study highlighted that early physical therapy not only aids in improving motor performance but also enhances activities of daily living (ADLs) capabilities (Hiraoka et al., 2017). Moreover, the integration of sensory reeducation techniques can help address the sensory deficits often observed in these patients, further contributing to their rehabilitation success, sensory stimulation technique involves using various modalities (auditory, visual, tactile) to enhance sensory recovery (Chen et al., 2021).

This case report aims to detail the physiotherapy interventions employed in a patient with thalamic-capsular hematoma complicated by perilesional oedema. By documenting the rehabilitation process and outcomes, we hope to contribute to the growing body of evidence supporting the importance of targeted physiotherapy in the recovery from thalamic injuries. The insights gained from this case may offer valuable guidance for clinicians managing similar cases in the future.

## Patients’ information

A 45-year-old female patient was alright 12 days back when, while brushing her teeth on 26th November at 8 am, she experienced sudden weakness in the left side of her body; she was taken immediately to a local hospital via auto in Chandrapur, where a plain CT of the brain was taken which suggested of right thalamic-capsular hematoma with perilesional oedema. She was admitted immediately and managed conservatively. Due to financial issues, she was brought to AVBRH on 4th November at 9 am, where she was admitted to neuro ICU immediately. The plain CT of the brain was advised by the neurologist and she was managed conservatively. She also has a medical history of chronic kidney disease and hypertension for 2 years. Neuro physiotherapy reference was given on 5th November 2024. She was catheterized on 6th November. For further rehabilitation, the patient was referred. Following referral to the physiotherapy department to improve patient functional independence and gain functional recovery (timeline as summarised in Table 1).

**Table 1:** Timeline of events.

Date	Events
26/11/2024	Date of the incident
04/12/2024	She was brought to AVBRH
05/12/2024	Neuro physiotherapy reference was initiated

## Timeline of events

### Physical and clinical findings

Before the examination, informed consent was obtained. It was noted that a Ryles tube, Foley catheter, IV line, and hemodialysis catheter (present since December 7th) were in place. A thorough neurological examination was performed. The patient was in an obtunded state and was disoriented to time, place, and person, with affected calculation, memory, and attention, and hemodynamically unstable. Motor examination indicated decreased muscle tone in the left-side upper and lower limbs as per the Tone Grading Scale (TGS) it revealed hypotonia in the left upper and lower limbs, and reflexes were diminished for the left side as shown in Table 2. The patient could not roll over in bed, sit, or stand independently.

**Table 2:** Presentation of reflexes.

Date	Biceps jerk		Triceps jerk		Supinator jerk		Knee jerk		Ankle jerk		Plantar
	Rt	Lt	Rt	Lt	Rt	Lt	Rt	Lt	Rt	Lt	
05/12/2024	++	+	++	+	++	+	+	++	++	+	Mute

### Diagnostic assessment

**Figure 1:**
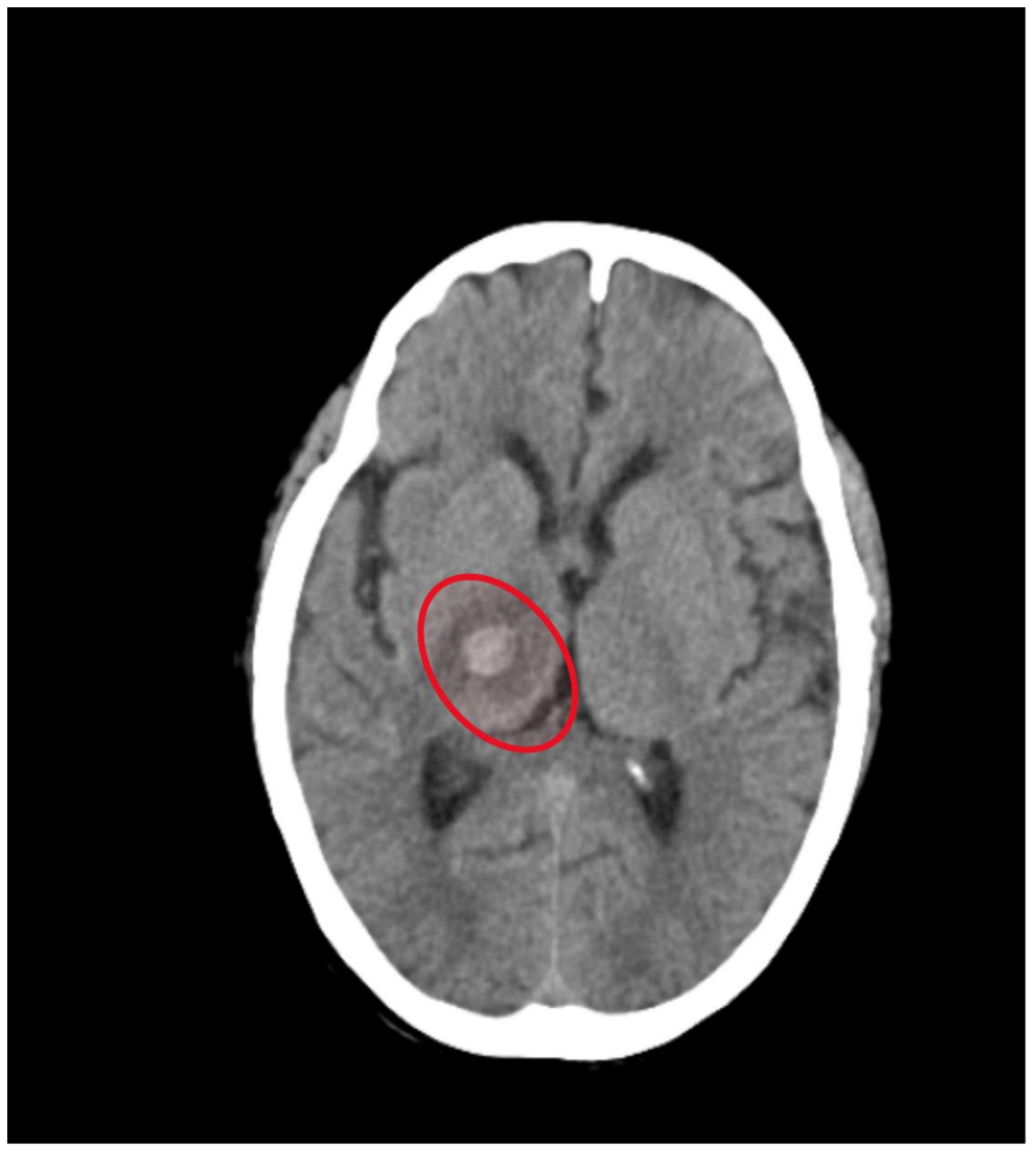
CT brain (plain).

**Figure 2:**
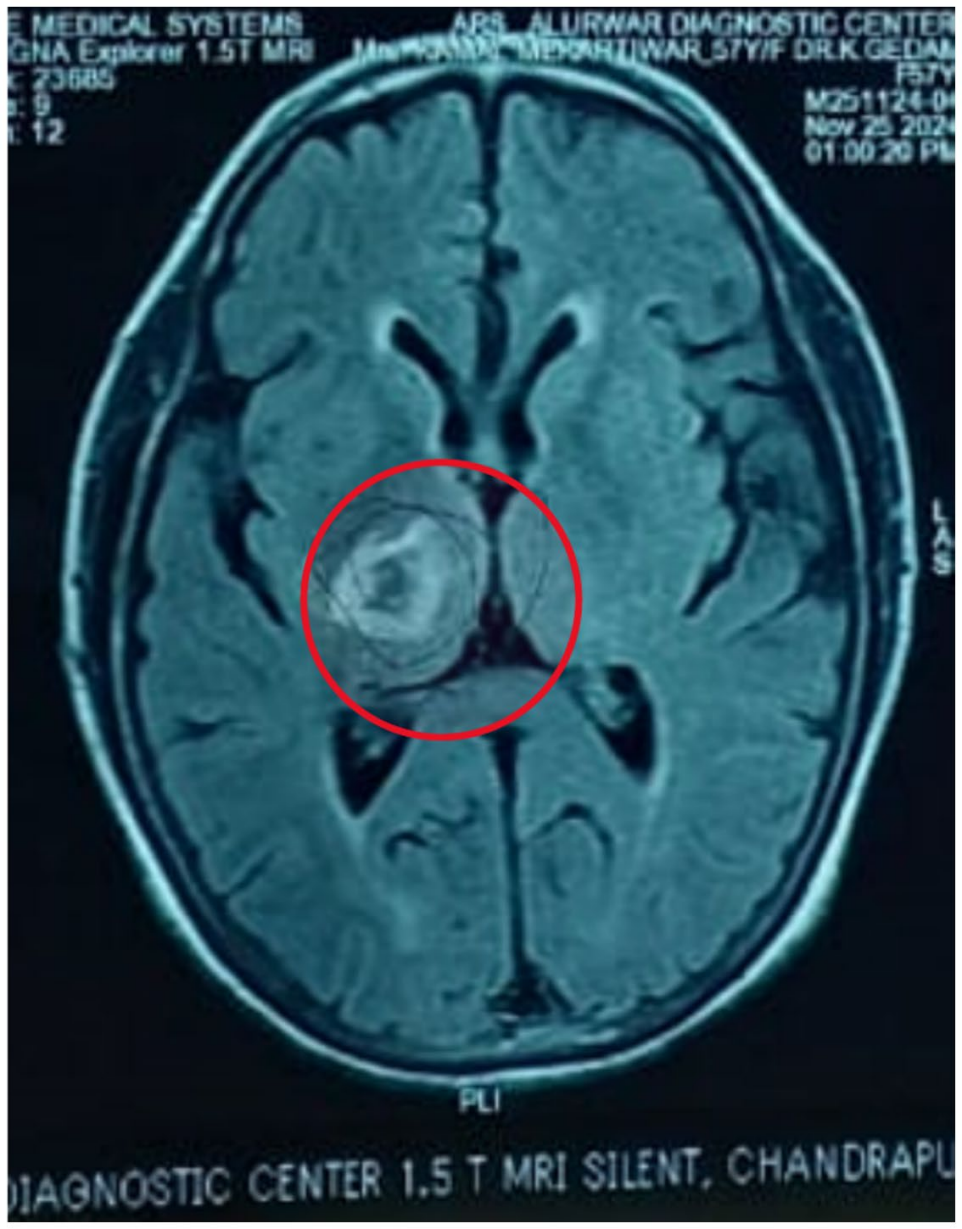
MRI brain.

Figures 1, 2 shows acute intraparenchymal haemorrhage with perilesional edema in the right thalamus and chronic lacunar infarct in pons (see Table 3).

**Table 3:** Clinical timeline of events demonstrated using the outcome measures.

Date	ICU mobility score	FIM	Galvenstein orientation and amnesia test (GOAT)	MMSE	Ranchos Los Amigos LOCF-revised
05/12/2024	0/10	18/126	0/100	17/30	Level 5 (confused inappropriate)
16/12/2024	0/10	31/126	60/100	21/30	Level 6 (confused appropriate)
01/01/2025	3/10	78/126	92/100	28/30	Level 7 (automatic inappropriate)

## Follow-up and outcome measures

### Therapeutic intervention

Table 4 shows physiotherapy intervention according to the patient problem list. After obtaining an informed consent from the patient’s family, a goal-oriented physiotherapy protocol was designed for a period of 5 weeks. The therapeutic interventions were performed by the same physiotherapist who assessed the patient. There were no complications in the 5 weeks of intervention. The patient did not receive any other interventions like speech therapy and occupational therapy, but the diet of the patient was given accordingly.

**Table 4:** Structured physiotherapy management aligned with the patient’s rehabilitation needs.

Goal	Management	Dosage	Rationale
Family and caregiver counselling and education	Proper education about the condition along with the importance of involvement of the family in every stage of the recovery. The importance of physiotherapy in its recovery is also to be highlighted		Educating and counselling families and caregivers in physiotherapy is essential for ensuring consistent care at home, improving patient compliance, and preventing complications. It empowers caregivers to support recovery effectively, leading to better rehabilitation outcomes and emotional well-being for the patient
To prevent any secondary complications like bedsores, pressure sores, joint contractures, etc.	Proper positioning techniques according to left hemiplegia also make sure that the head is 30 degrees elevated and, in the midline, and passive mobility exercises	Every 2 h initially, later 3–4 hourly	It helps optimize body alignment, prevent complications like pressure sores and contractures, and support effective breathing and circulation. It also enhances comfort and promotes functional movement during recovery
To enhance the level of consciousness	Multimodal stimulation Visual-using flashlight, past family or friends’ pictures, and auditory-by providing music and audio recordings of family or friends. Proprioception-PROM Intermittent theta burst stimulation	20 min for 3 h per day Every 10 s for a total of 192 s with 600 pulses (once a day)	It engages multiple senses to enhance neural activation, promoting better motor learning and cognitive development. This approach is especially beneficial in neuro-rehabilitation, as it supports more effective and integrated recovery
For motor recovery	Direct transcranial magnetic stimulation	Intermittent theta burst stimulation (iTBS) 2 s trains of bursts were repeated every 10 s for a total of 192 s with 600 pulses (once a day)	It is a non-invasive brain stimulation technique that enhances cortical excitability and promotes neuroplasticity. It supports motor recovery and functional improvements, particularly in neurological conditions like stroke
To improve the muscular strength	Strengthening of left-sided upper and lower limbs	10 reps * 1 set (2–3 times a day)	It helps improve muscle power, reduce weakness from disuse or neurological deficits, and enhance functional independence
To improve functional independence	Functional training (bed mobility training, sit-to-stand, training → standing → ambulation and gait training)	10 reps * 3 set	Enhances a patient’s ability to reposition independently, reducing the risk of pressure sores and respiratory complications. It also builds the foundation for transfers and mobility, promoting overall functional independence
To improve the tone of the muscles on the left side	Rood’s facilitatory approach- Fast tapping (over biceps tendon and quadriceps tendon) & joint compression (NDT)	10 reps * 1set (2 times per day)	To activate muscle responses and enhance motor function. Also, it focuses on guided movement and postural control to improve functional motor patterns, especially in individuals with neurological impairments

## Discussion

The management of thalamic hematomas requires a comprehensive, multidisciplinary approach, often combining medical treatment and surgical interventions. In recent years, the importance of physiotherapy in the rehabilitation phase has been increasingly emphasized due to its significant role in enhancing functional outcomes and improving patients’ quality of life following injury (Arboix et al., 2007).

The improvement observed in the patient can be attributed to the promotion of neuroplasticity and enhanced cortical reorganization. Transcranial magnetic stimulation, particularly intermittent theta burst stimulation (iTBS), has been shown to modulate cortical excitability and facilitate the restoration of disrupted neural circuits, thereby promoting motor recovery in stroke and hemorrhagic brain injuries (Di Lazzaro et al., 2023). Additionally, multimodal sensory stimulation—engaging multiple senses simultaneously—likely reinforced synaptic connections and helped improve interhemispheric communication, a critical factor in regaining motor and sensory function after thalamic injury (Todhunter-Brown et al., 2014). Rood’s approach, utilizing techniques such as tactile stimulation, joint compression, and muscle tapping, provided proprioceptive input that facilitated normalized muscle tone and voluntary movement, supporting spontaneous functional recovery (Ang et al., 2015).

Intervention methods were carefully selected and applied based on the patient’s presentation. TMS was administered over the ipsilesional motor cortex following established safety protocols to enhance motor cortical excitability (Langhorne et al., 2011; Rossini et al., 2015). Multimodal stimulation involved the integration of visual, auditory, and tactile tasks into physiotherapy sessions to maximize sensory feedback and motor planning. Rood’s techniques were applied by combining quick stretches and brushing to facilitate flaccid muscles, along with sustained pressure to inhibit spasticity when present. Each technique was tailored dynamically according to the patient’s daily responsiveness and fatigue levels.

The involvement of a multidisciplinary team significantly influenced the positive outcomes. Collaboration among physiotherapists, neurologists, rehabilitation physicians, occupational therapists, and speech therapists ensured comprehensive care. The team approach allowed for early detection and management of complications such as spasticity, shoulder subluxation, and cognitive deficits, thereby preventing secondary impairments and promoting holistic recovery (Lefaucheur et al., 2020). Regular interdisciplinary meetings and treatment plan adjustments according to the patient’s progress enhanced therapy efficacy.

Several precautions were essential to ensure patient safety and optimize rehabilitation outcomes. During TMS, strict adherence to established guidelines minimized the risk of seizures or adverse neurological effects (Langhorne et al., 2011). Close monitoring during multimodal stimulation sessions prevented overstimulation, fatigue, and frustration, which could hinder neuroplastic changes. When applying Rood’s techniques, it was crucial to avoid excessive or inappropriate sensory input that could provoke maladaptive responses such as spasticity or autonomic dysregulation. Furthermore, early mobilization strategies were employed carefully to avoid musculoskeletal complications such as contractures or pressure ulcers.

### Limitations and future scopes

The study was done on a single case for a short period of time. Future scope should focus on conducting prospective study using a large sample size incorporating this protocol to a large sample.

## Conclusion

This case study shows how crucial physical therapy is for a patient’s comeback after a thalamic-capsular bleed made worse by swelling around the injury. The patient saw big improvements in movement, sensation, and overall independence thanks to targeted treatments like functional rehab, muscle building normalizing muscle tone, and sensory work. Advanced methods like intermittent theta burst stimulation (iTBS) helped brain rewiring and functional recovery.

To get the best rehab results, you need personalized physical therapy and teamwork across different medical fields. This case gives doctors valuable lessons for treating similar brain problems and highlights why quick physical therapy is key in managing thalamic bleeds.

## Data Availability

The original contributions presented in the study are included in the article/supplementary material, further inquiries can be directed to the corresponding author.
